# Involvement of Cyclic Guanosine Monophosphate-Dependent Protein Kinase I in Renal Antifibrotic Effects of Serelaxin

**DOI:** 10.3389/fphar.2016.00195

**Published:** 2016-07-12

**Authors:** Veronika Wetzl, Elisabeth Schinner, Frieder Kees, Franz Hofmann, Lothar Faerber, Jens Schlossmann

**Affiliations:** ^1^Department of Pharmacology and Toxicology, University of RegensburgRegensburg, Germany; ^2^Novartis Pharma GmbHNuremberg, Germany; ^3^Institute of Pharmacology and Toxicology, Technical University of MunichMunich, Germany

**Keywords:** Relaxin, serelaxin, cGMP-dependent protein kinase, kidney, interstitial fibrosis, signaling, nitric oxide

## Abstract

**Introduction:** Kidney fibrosis has shown to be ameliorated through the involvement of cyclic guanosine monophosphate (cGMP) and its dependent protein kinase I (cGKI). Serelaxin, the recombinant form of human relaxin-II, increases cGMP levels and has shown beneficial effects on kidney function in acute heart failure patients. Antifibrotic properties of serelaxin are supposed to be mediated via relaxin family peptide receptor 1 and subsequently enhanced nitric oxide/cGMP to inhibit transforming growth factor-β (TGF-β) signaling. This study examines the involvement of cGKI in the antifibrotic signaling of serelaxin.

**Methods and Results:** Kidney fibrosis was induced by unilateral ureteral obstruction in wildtype (WT) and cGKI knock-out (KO) mice. After 7 days, renal antifibrotic effects of serelaxin were assessed. Serelaxin treatment for 7 days significantly increased cGMP in the kidney of WT and cGKI-KO. In WT, renal fibrosis was reduced through decreased accumulation of collagen1A1, total collagen, and fibronectin. The profibrotic connective tissue growth factor as well as myofibroblast differentiation were reduced and matrix metalloproteinases-2 and -9 were positively modulated after treatment. Moreover, Smad2 as well as extracellular signal-regulated kinase 1 (ERK1) phosphorylation were decreased, whereas phosphodiesterase (PDE) 5a phosphorylation was increased. However, these effects were not observed in cGKI-KO.

**Conclusion:** Antifibrotic renal effects of serelaxin are mediated via cGMP/cGKI to inhibit Smad2- and ERK1-dependent TGF-β signaling and increased PDE5a phosphorylation.

## Introduction

Kidney fibrosis is a key contributor to CKD, mainly resulting from diabetes or hypertension in developed countries. The prevalence of CKD is estimated 7.2% in patients aged 30 years or older (Zhang and Rothenbacher, 2008). Renal fibrosis is characterized by excessive accumulation of ECM including collagen and fibronectin. TGF-β and CTGF are profibrotic cytokines which promote fibroblast to myofibroblast differentiation expressing α-smooth muscle actin (α-SMA). These cells are predominantly expressed in fibrotic tissue contributing to the deposition of ECM and modulation of MMPs. To prevent or reduce renal fibrotic tissue, the modulation of cyclic nucleotides, particularly cGMP might be promising. cGMP has already shown to be increased in kidney fibrosis as well as further increased through pharmacological intervention for the amelioration of kidney fibrosis and improvement of renal function (Wang et al., 2006). Currently, cGMP modulation is achieved by several therapeutic approaches including NO donors and soluble guanylate cyclase (sGC) stimulators (Schlossmann and Schinner, 2012).

Relaxin was firstly described by Hisaw (1929) due to its antifibrotic effects in the reproductive system. By now, RLX showed pleiotropic effects in several experimental and clinical research, mainly mediated through its G-protein coupled receptor RXFP1 (Bathgate et al., 2013). Antifibrotic effects involve NO, sGC and the downstream mediator cGMP to inhibit TGF-β signaling (Samuel, 2005; Halls et al., 2015; Wang et al., 2016). RLX – the recombinant form of the naturally occurring human pregnancy hormone relaxin-II – is a cGMP modulating agent, which is currently being tested in a phase III clinical trial for acute heart failure. About 18% to 40% of patients with acute heart failure also experience worsening of renal function during acute decompensation, which adversely affects prognosis (Cole et al., 2012). Serelaxin has already shown improved organ function as indicated by reduced biomarker levels for renal damage after recompensation (Metra et al., 2013; Teerlink et al., 2013).

Schinner et al. (2013) and Cui et al. (2014) have demonstrated renal antifibrotic signaling via NO/cGMP and the cGMP-dependent protein kinase I (cGKI) in rodents after the administration of NO donors and soluble guanylyl cyclase stimulators. However, a possible role of cGKI in RLX’s antifibrotic effect still remains unclear.

Further research is necessary to elucidate downstream mechanisms involved in the RXFP1- NO/cGMP-dependent antifibrotic pathway. The aim of this study was to examine the antifibrotic-signaling pathway of RLX in the kidney. We hypothesized that RLX’s antifibrotic properties are mediated through cGKI.

## Materials and Methods

### Animals

129/Sv-WT and 129/Sv-cGKI-KO mice (Pfeifer et al., 1998) were bred and maintained in the animal facilities of University of Regensburg. Experiments are conducted according to the guide for the Care and Use of Laboratory Animals published by the US National Institute of Health. Protocols were approved by local authorities for animal research (Regierung der Oberpfalz, Bayern, Germany, #54-2532.1-26/13) and conducted according to German law for animal care.

### UUO and RLX Treatment

Unilateral ureteral obstruction, an established mouse model for chronic renal interstitial fibrosis (Chevalier et al., 2009), was performed according to Schinner et al. (2013) Kidney tissues of WT and cGKI-KO mice were divided into four groups: mice untreated or treated with RLX. Fibrotic tissue was derived from UUO-obstructed kidney, healthy tissue from contralateral kidney served as control. RLX was diluted in 20 mM sodium acetate (pH = 5) and administered continuously through osmotic minipumps (Alzet; model 1007D) immediately after UUO for 7 days (0.5 mg/kg/day).

### Tissue Preparation

Under isoflurane inhalation kidney tissue was removed after perfusion with 0.9% NaCl 7 days after UUO. Proteins from kidney tissue were solubilized in 50 mM Tris/2% SDS/phosphatase inhibitor (PhosSTOP, Roche; 1 tablet/5 ml)/protease inhibitors (leupeptin 0.5 μg/ml, PMSF 300 μM, benzamidine hydrochlorid 1 mM, and EDTA 5 mM) for 45 min at 7°C followed by centrifugation at 12400*g*, 7°C, 45 min. In supernatants, protein content was determined by modified Lowry method (Lowry et al., 1951) and stored at –80°C until analysis.

### Western Blot Analysis

Protein expression of GAPDH, ERK1/2, P- ERK1/2, P-Smad2, P-VASP (Ser 239), GAPDH (antibodies from Cell Signaling, Danvers, MA, USA), TGF-β, CTGF, PDE5a (antibodies from Santa Cruz Biotechnology, Heidelberg, Germany) and P-PDE5a (Ser 92) (FabGennix, Frisco, TX, USA) were assayed by western blotting. After SDS-PAGE proteins were transferred to PVDF membranes. Donkey anti-goat IgG HRP (santa cruz Biotechnology, Heidelberg, Germany) and donkey anti-rabbit IgG HRP (Dianova GmbH, Hamburg, Germany) were used as secondary antibodies. Quantification was performed by ImageLab™ densitometry software (BioRad, München, Germany). Values were related to corresponding GAPDH values, except P-ERK1/2 is related to ERK1/2. Change of markers from healthy to fibrotic tissue was compared by values of fibrotic tissue in relation to healthy tissue, both untreated WT mice. The influence of treatment on markers was determined only in fibrotic tissue by analyzing values of markers in relation to mean values of untreated fibrotic WT, which were set as 1.

### Gelatin Zymography Assay

The activity of MMP2 and MMP9 was determined using gelatin zymography. SDS-PAGE was performed with a gel containing 0.1% gelatin. After washing (100 mM NaCl and 2.5% Triton X-100 in 50 mM Tris-HCl, pH 7.5) the gel was transferred to a reaction buffer (200 mM NaCl, 0.02% NaN_3_, 0.5 μM ZnCl_2_, 1 mM CaCl_2_, 2% Triton-X 100, in 50 mM Tris-HCl, and pH 7.5) for enzymatic reaction at 37°C overnight. Gel was stained with Coomassie blue, destained in 10% acetic acid (*v*/*v*), and 30% methanol (*v*/*v*) and quantified using Image Lab. MMPs in fibrotic tissue were expressed as relative values of markers in fibrotic kidneys from untreated WT mice.

### Enzyme-Linked Immunosorbent Assays

Serelaxin serum levels were determined using Human Relaxin-2 Quantikine^®^ ELISA (R&D Systems, Wiesbaden-Nordenstadt, Germany). cGMP levels in kidney tissue using cGMP EIA (IBL-Cayman, Hamburg, Germany). Measurements were performed according to manufacturer’s instructions.

### Sirius Red/Fast Green Method for Quantification of Collagen

Collagen levels in the kidneys were measured by a modified sirius red/fast green method (Lopez-De Leon and Rojkind, 1985), based on selective binding of sirius red to collagen and fast green dye binding to non-collagen proteins. Sirius red/fast green staining was calculated as increase (%) of collagen [ratio collagen/non-protein collagen] after 7 days UUO related to healthy kidney as described previously (Schinner et al., 2013).

### Immunohistochemistry

Kidney tissues were cut at 4 μm. Immunohistochemistry and quantification was performed according to Schinner et al. (2013) Primary antibodies are mouse anti-α-SMA (Beckman Coulter, Krefeld, Germany), rabbit anti-Col1a1 and rabbit anti-fibronectin (Abcam, Cambridge, UK). Alexa 647-conjugated donkey anti-rabbit and Cy2-conjugated donkey anti-mouse served as secondary antibodies. For quantification the increase after UUO was related to the healthy kidney.

### Quantitative RT-PCR

Isolation of total RNAs from kidney tissue, determination using quantitative RT-PCR as well as calculation was described previously (Schinner et al., 2013). mRNA levels of αSMA, fibronectin, Col1a1, MMP2, and MMP9 were detected. 18S rRNA served as housekeeping gene. The ΔΔ*C*_T_ (cycle threshold) value is calculated from the difference of the corresponding control (C) and fibrosis-induced kidneys (F) [ΔΔ*C*_T_ = Δ*C*_T_(C) - Δ*C*_T_(F)]. Then, the ratio of expression (*r*) was determined [r = 2^ΔΔCT^].

### Serum Creatinine

Serum creatinine was determined by a previously published HPLC method with minor modifications (Schramm et al., 2014). Serum (10 μl) was mixed with 50 μl perchloric acid to precipitate proteins. The tube was mixed, kept at 4°C for 15 min, then centrifuged (5 min, 10,800 g). 5 μl of the supernatant was injected into the HPLC apparatus (Prominence LC20 series equipped with a LC20A photometric detector set at 234 nm; Shimadzu, Duisburg, Germany). Separation was performed using a Zorbax 300-SCX 5 μm, 150 × 4.6 mm, analytical column (Agilent, Waldbronn, Germany) and a mobile phase consisting of 5 mM sodium acetate (pH = 5.1)/acetonitrile (800:200, *v:v*). Creatinine eluted after 6.3–6.5 min at a flow rate of 1.0 ml/min (column temperature 35°C).

### Statistical Analysis

All data are expressed as mean ± SEM. Statistical differences between two means were calculated by unpaired student’s *t*-test (two-tailed, confidence interval 95%). Statistical significance was marked by asterisks (^∗^*p* < 0.05; ^∗∗^*p* < 0.01; ^∗∗∗^*p* < 0.001). *n* indicates number of animals. For data analysis, GraphPad Prism, version 6 (GraphPad software, Inc., La Jolla, CA, USA), was used.

## Results

### Effect of RLX on cGMP and cGKI in Kidney Tissue

Plasma levels of relaxin were determined through RLX ELISA in WT and cGKI-KO mice. In untreated mice serum levels were at 11.4 ± 1.4 pg/ml for WT (*n* = 11) and at 0.04 ± 0.04 pg/ml for cGKI-KO (*n* = 5). In RLX-treated mice serum levels increased to 20361 ± 2290 pg/ml for WT (*n* = 14) and 13357 ± 3122 pg/ml for cGKI-KO mice (*n* = 4, not significantly different for WT and cGKI-KO), which was highly statistically significant compared to untreated mice (WT: *p* < 0.001; cGKI-KO: *p* = 0.0018).

**FIGURE 1:**
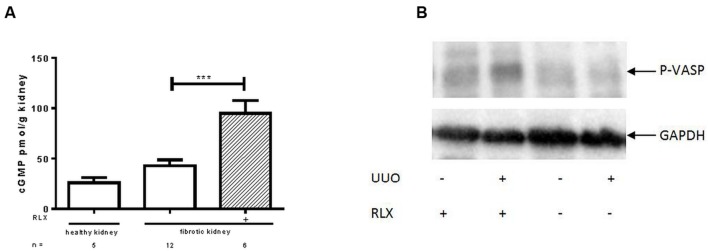
**Determination of (A) cGMP levels and (B) protein kinase activity of cGKI, determined by VASP phosphorylation (Ser239) in healthy or fibrotic kidney tissue of WT mice 7 days after UUO; mice were untreated or treated with serelaxin for 7 days during UUO.** As described in methods increase of renal cGMP levels was quantified with ELISA according to manufacturer’s instructions. VASP phosphorylation at Ser 239 was determined by western blotting (50 μg protein) after normalization to GAPDH; cGMP, cyclic guanosine monophosphate; GAPDH, glyceraldehyde-3-phosphate dehydrogenase; P-VASP, phospho-vasodilator- stimulating phosphoprotein; RLX, serelaxin; UUO, unilateral ureteral obstruction; ^∗∗∗^*p* < 0.001.

cGMP levels were investigated in kidney tissue. **Figure 1A** shows that cGMP was higher in fibrotic tissue 7 days after UUO than in the contralateral kidney (42.8 ± 5.7 vs. 25.9 ± 5.2 pmol/g). After RLX treatment cGMP levels of fibrotic kidneys further increased significantly (95.0 ± 12.5 pmol/g).

The activity of cGKI can be determined by quantification of cGKI-specific VASP phosphorylation at Serine 239. In **Figure 1B** an increase of VASP phosphorylation was observed after RLX treatment, in healthy and in fibrotic WT tissues. The increase in WT mice, expressed as relative values in kidneys from untreated WT mice, changed from 0.94 ± 0.12 (*n* = 5) to 1.68 ± 0.32 (*n* = 5) after treatment. In cGKI-KO this effect was lacking (0.79 ± 0.22; *n* = 3 vs. 0.86 ± 0.17; *n* = 2).

### Effect of RLX on α-SMA in WT- and cGKI-KO Kidneys

**FIGURE 2:**
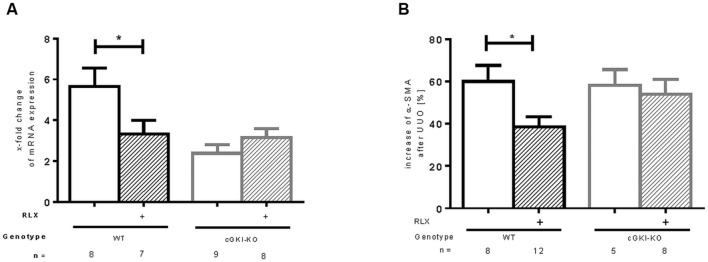
**Effect of serelaxin on (A) mRNA and (B) protein expression of α-SMA in kidney tissue of WT and cGKI-KO mice.** mRNA was determined with RT-qPCR and 18s rRNA as housekeeping gene. Results are shown as fold change of mRNA expression (2^ΔΔct^) in the fibrotic kidney relative to contralateral healthy kidney, which was set as 1. The increase of α-SMA protein expression [%] in fibrotic renal tissue compared to contralateral kidney was quantified by immunohistochemistry; α-SMA, α-smooth muscle actin; cGKI, cGMP-dependent protein kinase I; KO, knockout; RLX, serelaxin; UUO, unilateral ureteral obstruction; WT, wildtype, ^∗^*p* < 0.05.

mRNA of α-SMA, a marker of myofibroblast differentiation, (Nagamoto et al., 2000) was increased in both WT and cGKI-KO after UUO. A reduction of mRNA was observed after treatment with RLX in WT, whereas no effect was seen in cGKI-KO after treatment (**Figure 2A**). As expected, in unobstructed renal tissue only vascular smooth muscle cells were immunostained with α-SMA, in UUO-obstructed kidneys enhanced interstitial expression was observed (data not shown). In WT, α-SMA protein was elevated compared to the contralateral kidney, after RLX treatment the increase was significantly reduced. In cGKI-KO no significant reduction of α-SMA protein expression through RLX was demonstrated (**Figure 2B**).

### Effect of RLX on ECM Accumulation in WT- and cGKI-KO Kidneys

**FIGURE 3:**
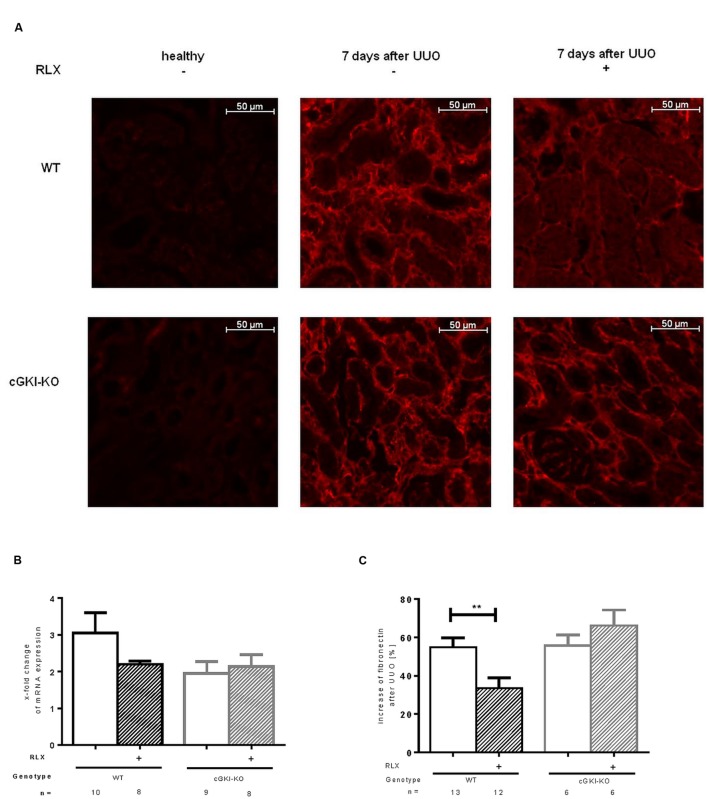
**(A)** Immunohistochemical staining of fibronectin in kidney tissue of WT and cGKI-KO mice – healthy or 7 days after UUO treated or untreated with serelaxin; effect of serelaxin on **(B)** mRNA and **(C)** protein expression of fibronectin in kidney tissue of WT and cGKI-KO mice; mRNA was determined with RT-qPCR and 18s rRNA as housekeeping gene. Results are shown as fold change of mRNA expression (2^ΔΔct^) in the fibrotic kidney relative to contralateral healthy kidney, which was set as 1. The increase of fibronectin protein expression [%] in fibrotic renal tissue compared to contralateral kidney was quantified by immunohistochemistry; cGKI, cGMP-dependent protein kinase I; KO, knock out; RLX, serelaxin; UUO, unilateral ureteral obstruction; WT, wildtype. ^∗∗^*p* < 0.01.

**FIGURE 4:**
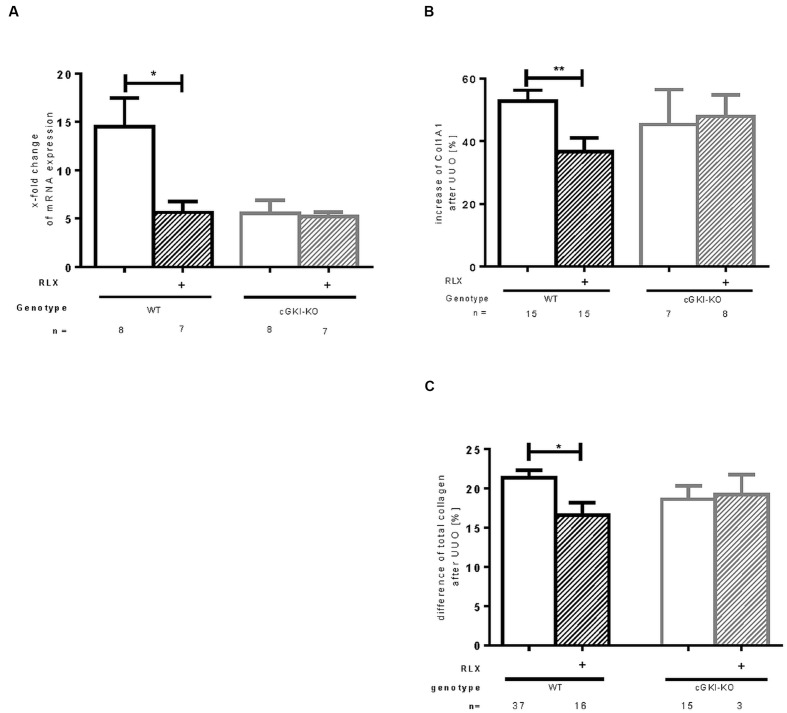
**Effect of serelaxin on (A) mRNA and (B) protein expression of Col1A1 as well as (C) protein expression of total collagen in kidney tissue of WT and cGKI-KO mice.** mRNA was determined with RT-qPCR and 18s rRNA as housekeeping gene. Results are shown as fold change of mRNA expression (2^ΔΔct^) in the fibrotic kidney relative to contralateral healthy kidney, which was set as 1. The increase of Col1A1 protein expression [%] in fibrotic renal tissue compared to contralateral kidney was quantified by immunohistochemistry; the increase of total collagen [%] was quantified with sirius red/fast green method; cGKI, cGMP-dependent protein kinase I; Col1A1, collagen1A1; KO, knock out; RLX, serelaxin; UUO, unilateral ureteral obstruction; WT, wildtype. ^∗^*p* < 0.05, ^∗∗^*p* < 0.01.

Fibronectin and Col1A1 are components of ECM, whose gene expressions are upregulated in fibrosis through TGF-β signaling. mRNA of both genes were elevated in fibrotic WT kidney 7 days after UUO compared to the contralateral kidney. Fibronectin and Col1A1 mRNA were reduced through RLX in WT (**Figures 3B and 4A**). Protein expression was strongly elevated by more than 40% for fibronectin and Col1A1 in both WT and cGKI-KO mice, compared to kidneys without UUO (for fibronectin, **Figures 3A,C**). Significant reduction of both proteins through RLX treatment was observed only in WT after 7 days of treatments.

Protein expression of total collagen was elevated by 21.4% ± 0.99 in fibrotic WT and significantly decreased through RLX treatment to an elevation of 16.6% ± 1.7 compared to unobstructed kidneys (*p* = 0.0126). In cGKI-KO, effects of RLX on ECM accumulation were not observed (**Figures 3A,C** and **4B,C**).

### Regulation of MMPs by RLX in WT- and cGKI-KO Kidneys

**FIGURE 5:**
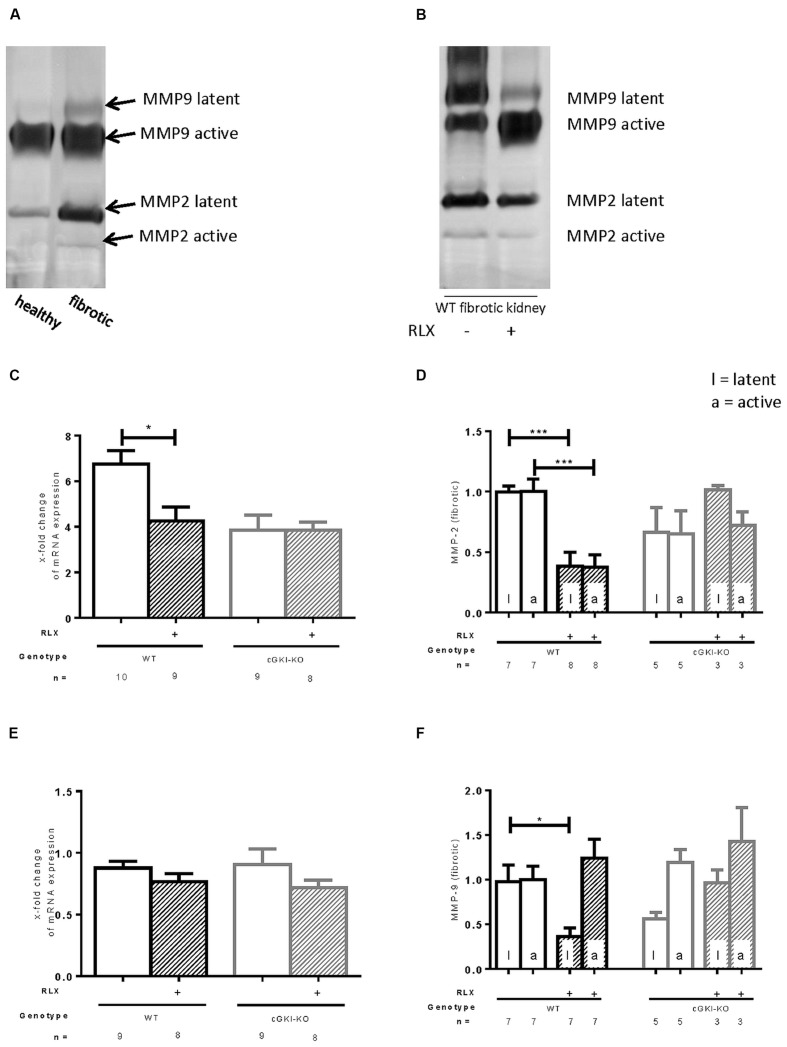
**Effect of serelaxin on (C), (E) mRNA and (A), (B), (D), (F) protein expression of MMP-2, MMP-9 determined by zymography (each 70 μg protein) in kidneys from WT and cGKI-KO mice. (A)** Representative blot from untreated WT, which illustrates active and latent MMP expression in healthy and fibrotic renal tissue; **(B)** representative blot from fibrotic WT kidneys untreated or treated with serelaxin; mRNA was determined with RT-qPCR and 18s rRNA as housekeeping gene. Results are shown as fold change of mRNA expression (2^ΔΔct^) in the fibrotic kidney relative to contralateral kidney, which was set as 1 [**(C)** MMP2; **(E)** MMP9]; latent and active MMP expression determined by zymography (70 μg protein) and is shown only in fibrotic tissue. Each value of WT and cGKI-KO is related to untreated fibrotic WT, which was set as 1 [**(D)** MMP2; **(F)** MMP9]; cGKI, cGMP-dependent protein kinase I; KO, knock out; MMP, matrix metalloproteinase; RLX, serelaxin; UUO, unilateral ureteral obstruction gibt es nicht; WT, wildtype, ^∗^*p* < 0.05, ^∗∗∗^*p* < 0.001.

MMPs are relevant for the degradation of ECM. mRNA of MMP2 was 6.8-fold (± 0.58) increased in fibrotic tissue, whereas MMP9 was not increased in that pathological condition (0.88-fold ± 0.53). Through RLX treatment, only the elevated levels of MMP2 mRNA were significantly decreased (4.3-fold ± 0.61), no changes were found for MMP9 mRNA after treatment (0.76-fold ± 0.067; **Figures 5C,E**). Zymography distinguishes between latent and active MMP proteins (**Figures 5A,B**). In WT, latent and active forms of MMP2 were elevated in fibrosis (5.9-fold ± 1.3; 4.2-fold ± 0.84), protein expression of latent MMP9 was significantly enhanced in fibrotic tissue (3.0-fold ± 0.42), active form significantly decreased (0.69-fold ± 0.098) compared to contralateral kidneys (*n* = 12, respectively).

**Figures 5D,F** show regulation of MMPs only in fibrotic tissue expressed as relative values of markers in kidneys from untreated WT mice. Consistent with data from mRNA, latent and active forms of MMP2 were both significantly reduced through RLX treatment in fibrotic kidneys. Through reduction of latent MMP9 and increase of active MMP9 after treatment physiological conditions were nearly restored.

The above described effects on MMP2 and MMP9 were not observed in cGKI-KO mice after treatment (**Figures 5D,F**).

### Signaling Molecules in WT- and cGKI-KO Kidneys Treated with RLX

**FIGURE 6:**
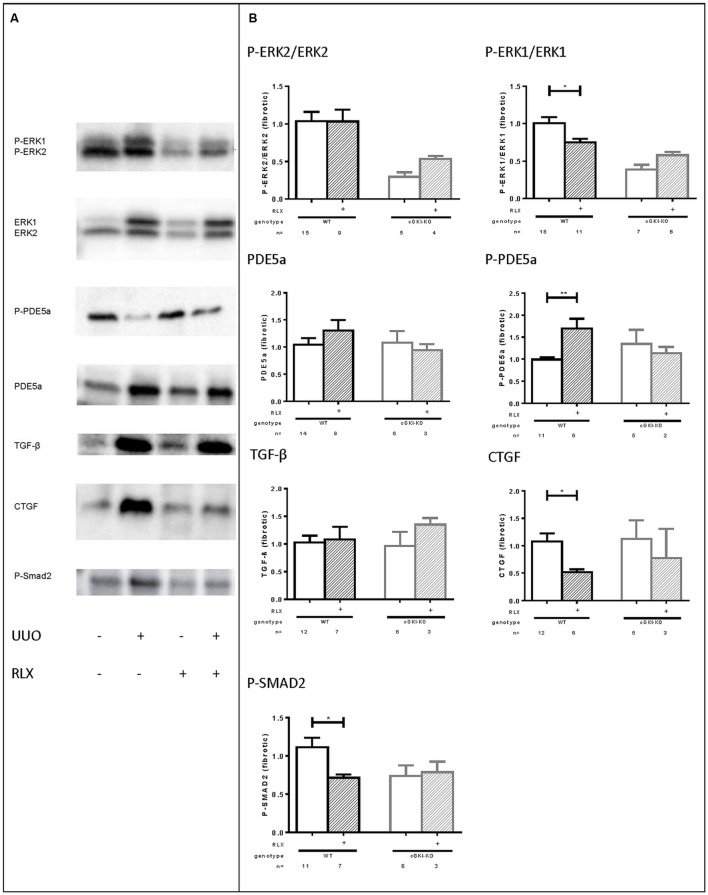
**(A)** Representative western blots (each 50 g protein) of signaling molecules in kidney tissue of WT mice with or without UUO untreated or treated with serelaxin; **(B)** effect of serelaxin on protein expression of signaling molecules (P-ERK2/ERK2, P-ERK1/ERK1, PDE5a, P-PDE5a, TGF-β, CTGF, P-SMAD2) in fibrotic kidney from WT and cGKI-KO mice; in **Figure 5 (B)** effects of serelaxin on protein expression of markers (normalized to GAPDH) was determined only in fibrotic tissue. Each value of WT and cGKI-KO is related to untreated fibrotic WT, which was set as 1; α-SMA, α-smooth muscle actin; cGKI, cGMP-dependent protein kinase I; cGMP, cyclic guanosine monophosphate; CTGF, connective tissue growth factor; ECM, extracellular matrix; eNOS, endothelial nitric oxide synthase; ERK, extracellular-signal regulated kinase; GAPDH, glyceraldehyde-3-phosphate dehydrogenase; GMP, guanosine monophosphate; KO, knock out; MMPs, matrix metalloproteinases; PDE5a, phosphodiesterase 5a; RLX, serelaxin; RXFP1, relaxin receptor; Smad, small mothers against decapentaplegic protein; TGF-β, transforming growth factor-β; UUO, unilateral ureteral obstruction; WT, wildtype, ^∗^*p* < 0.05, ^∗∗^*p* < 0.01.

As mentioned above, cGMP levels were elevated through RLX and, so far, cGKI-KO mice showed no antifibrotic effects, which suggested the involvement of the NO/cGMP/cGKI pathway in the antifibrotic effect of RLX. Subsequently, several signaling molecules were analyzed by western blotting, which are involved in fibrosis. Representative western blots demonstrated modulation of the selected markers in fibrotic conditions compared to healthy (**Figure 6A**).

Downstream to RLX, protein expression of phosphorylated ERK 1/2 (P-ERK1, P-ERK2) was analyzed. P-ERK1 was significantly elevated in kidneys of WT mice compared to the contralateral after undergoing UUO for 7 days (P-ERK1:1.8-fold ± 0.24; *n* = 7). cGMP is degraded by PDE5a, which is strongly upregulated in fibrosis (6.0-fold ± 1.3; *n* = 10), whereas phosphorylation of PDE5a at the cGMP-dependent phosphorylation site serine 92 (Thomas et al., 1990; 0.68-fold ± 0.12; *n* = 7) was decreased. TGF-β is a profibrotic cytokine, that was 5.3-fold (± 1.1; *n* = 12) elevated in the fibrotic kidneys compared to the contralateral renal WT tissue. Its downstream profibrotic signaling is dependent on Smad or -independent via ERK1/2 phosphorylation. Both P-Smad2 (1.926 ± 0.2384; *n* = 12) and ERK-1 (see above) were significantly elevated in fibrotic renal WT tissue. Additionally, further TGF-β transcription genes, e.g., CTGF were analyzed in this experiment. It was confirmed, that CTGF levels were elevated 3.3-fold (± 0.77; *n* = 12) in obstructed WT kidneys.

Protein levels of all signaling markers were significantly different in healthy and fibrotic kidney tissue from WT mice – P-PDE5a was reduced, remaining markers increased in fibrosis.

**Figure 6B** illustrates the influence of RLX on markers only in fibrotic tissue of WT and cGKI-KO. Values are related to untreated fibrotic WT kidneys, which were set as 1.

The phosphorylation of ERK1 normalized to total ERK1 was significantly reduced in WT through RLX treatment, but not in cGKI-KO.

eNOS and nNOS were increased in fibrosis, but were not further enhanced after treatment with RLX (data not shown).

The cGMP degrading PDE 5a is slightly enhanced through RLX in WT, and furthermore RLX significantly enhanced phosphorylation of PDE5a, indicating enhanced activity of PDE5a in fibrosis. The treatment-dependent increase in PDE5a phosphorylation is lacking in cGKI-KO. TGF-β itself remained unchanged in fibrotic tissue despite RLX treatment, but downstream signaling of TGF-β was affected through treatment. RLX significantly reduced P-Smad2 in WT, but not in cGKI-KO. Signaling via Smad-independent pathway was regulated through ERK1/2 phosphorylation (Leask and Abraham, 2004). As mentioned above, ERK1 phosphorylation was significantly reduced after treatment only in WT kidneys.

As previously stated, collagen, fibronectin and myofibroblast differentiation (α-SMA) was decreased (see above). 7 days after UUO continuous infusion of RLX decreased CTGF significantly in WT, but not in cGKI-KO.

### Effect of RLX on Kidney Function of WT- and cGKI-KO Mice

**FIGURE 7:**
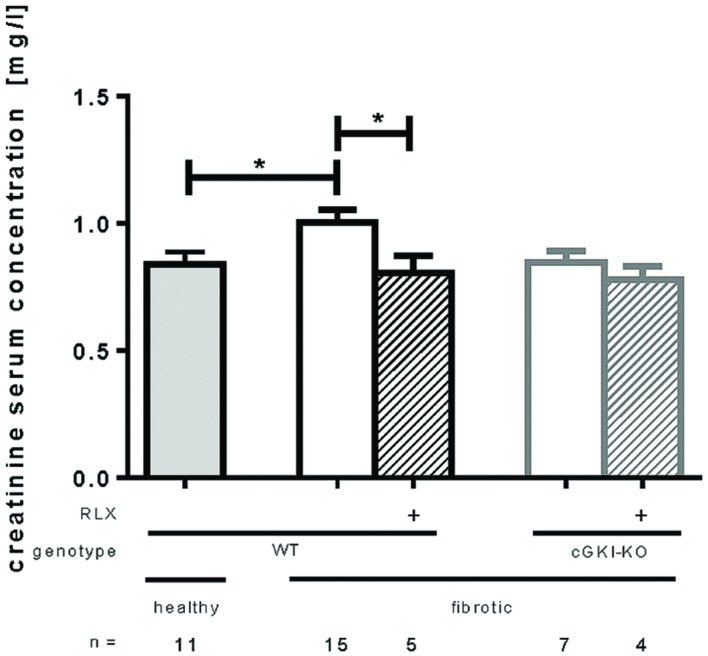
**Serum creatinine levels in healthy mice or 7 days after UUO in WT and cGKI-KO mice.** Serum creatinine levels were determined via high pressure liquid chromatography as described in methods. cGKI, cGMP-dependent protein kinase I; KO, knock out; RLX, serelaxin; WT, wildtype, ^∗^*p* < 0.05.

Kidney function was measured by serum creatinine levels analyzed 7 days after UUO. **Figure 7** shows that serum creatinine increased significantly after UUO, but renal performance improved significantly through RLX treatment by reducing serum creatinine levels from 1.0 mg/l ± 0.049 to 0.80 mg/l ± 0.069 in WT. cGKI-KO did not improve kidney function through RLX.

## Discussion

**FIGURE 8:**
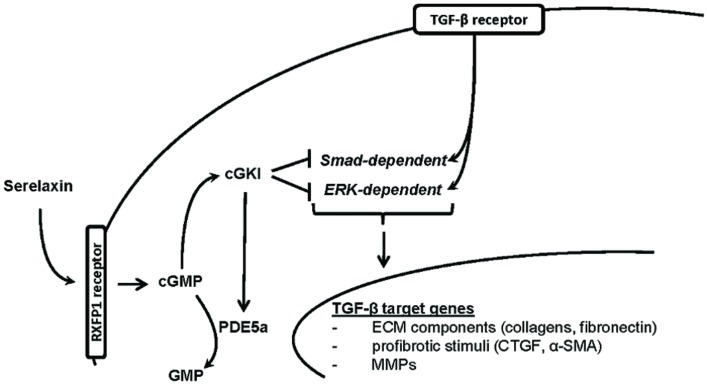
**Proposed hypothesis for *in vivo* antifibrotic signaling pathway of serelaxin in kidney.** Serelaxin mediates its antifibrotic effect via RXFP1/cGMP/cGKI to inhibit TGF-β dependent Smad- and ERK-phosphorylation, which subsequently decreases ECM accumulation and suppresses profibrotic stimuli; α-SMA, α-smooth muscle actin; cGKI, cGMP-dependent protein kinase I; cGMP, cyclic guanosine monophosphate; CTGF, connective tissue growth factor; ECM, extracellular matrix; ERK, extracellular-signal regulated kinase; GMP, guanosine monophosphate; MMPs, matrix metalloproteinases; PDE5a, phosphodiesterase 5a; RXFP1, relaxin family peptide receptor 1; Smad, small mothers against decapentaplegic protein; TGF-β, transforming growth factor-β.

In this study, we demonstrated that RLX mediated its antifibrotic effects via NO/cGMP/cGKI, to inhibit TGF-β signaling through Smad- and ERK1-dependent pathways (**Figure 8**).

Serelaxin modulated remodeling processes on several levels resulting in reduced ECM accumulation. Samuel et al. (2003) demonstrated that relaxin-1 deficient mice developed age-related fibrosis in the kidney and other organs. Endogenous relaxin was able to reduce early fibrotic changes in kidney tissue after UUO (Hewitson et al., 2007). RLX administration has already shown renal antifibrotic effects in models of kidney injury, including renal papillary necrosis (Garber et al., 2001), antiglomerular basement membrane model (McDonald et al., 2003), spontaneously hypertensive rats (Lekgabe et al., 2005), models of renal mass reduction (Garber et al., 2003), and UUO (Hewitson et al., 2010).

In our study, renal interstitial fibrosis was induced by UUO for 7 days, a method for rapid development of interstitial fibrosis with enhanced ECM deposition (Chevalier et al., 2009) In our experiments, ECM components and ECM-producing cells were increased. Signaling pathways were regulated differently in the fibrotic and unobstructed kidney tissue. TGF-β and CTGF, important profibrotic cytokines, were markedly elevated in fibrotic kidneys. TGF-β signaling is mediated via Smad-dependent or independent pathways to induce myofibroblast differentiation and gene expression of collagens, fibronectin, CTGF, ECM-degrading MMPs, and several other profibrotic stimuli (Leask and Abraham, 2004). In accordance with previous work on UUO (Masaki et al., 2003; Pat et al., 2005; Hewitson et al., 2010), in the current experiment both signaling mechanisms are activated in fibrotic tissues, indicated through enhanced ERK1 as well as Smad2 phosphorylation.

Animals were treated with RLX starting immediately after UUO. cGMP was upregulated in fibrotic tissue and further increased in mice treated with RLX, suspecting a cGMP-dependent-signaling mechanism for RLX. The NO/cGMP-signaling pathway has often been demonstrated to influence remodeling processes in different organs, including the kidney (Wang-Rosenke et al., 2011; Sun et al., 2012; Schinner et al., 2015). PDE inhibitors have already shown their antifibrotic effects by enhanced cGMP availability (Rodriguez-Iturbe et al., 2005; Bae et al., 2012). cGKI is involved in the signaling process of RLX, as RLX treated WT mice caused enhanced activity of cGKI, indicated by the cGKI-specific phosphorylation at Ser239 of VASP and Ser92 of PDE5 compared to untreated mice. The association of cGKI with antifibrotic effects was already shown by Schinner et al. (2013) and Cui et al. (2014 in a model of UUO-induced renal fibrosis. This was confirmed in our model for RLX signaling, as we observed a significant antifibrotic and antiremodelling effect indicated by reduced fibronectin, Col1A1, total collagen and α-SMA in WT. These effects were lacking in the cGKI-KO, suggesting an involvement of cGKI in the antifibrotic properties of RLX in the kidney. As previously observed (Schinner et al., 2013), fibrosis marker were reduced in non-treated cGKI-KO compared to non-treated WT; however, RLX treatment did not decrease fibrosis marker in cGKI-KO.

Serelaxin mainly realizes its antifibrotic effects via the relaxin receptor RXFP1, which is present or expressed in the kidney (Hsu et al., 2002; Halls et al., 2015) as well as in rat renal fibroblasts and myofibroblasts *in vitro* (Masterson et al., 2004) and *in vivo* (Mookerjee et al., 2009). The effect of RXFP1 on the enhancement of NO still remains unclear. It is discussed, that it signals through nNOS to enhance NO/cGMP in myofibroblasts (Mookerjee et al., 2009; Chow et al., 2012) without the involvement of eNOS. eNOS appears to have a more significant role in the arterial vasodilatory effect (McGuane et al., 2011) of RLX than in the antifibrotic. However, eNOS as well as nNOS were not increased in our experiment, this does not exclude a role of eNOS or nNOS in the protective effect of RLX as the interplay of the diverse NOS isoforms in the suppression against interstitial renal fibrosis was previously observed (Morisada et al., 2010). Changes in the expression levels do not reveal the alterations in the activity of these enzymes, e.g., an altered intracellular calcium concentration would change the activity of the calcium-dependent eNOS and nNOS. The inhibition of PDEs, e.g., the cGMP specific PDE5a, also augments cGMP. However, PDE5a was strongly enhanced in fibrosis, the phosphorylation of PDE5a at the cGKI-specific phosphorylation site serine 92 (Thomas et al., 1990) was additionally strongly upregulated after treatment. The phosphorylation implicates increased enzyme activity as well as augmented affinity of cGMP to allosteric-binding sites (Rybalkin et al., 2002; Francis et al., 2010) suggesting an autoregulatory feedback mechanism. Due to the cGKI-specific phosphorylation, no effect of RLX on PDE5a phosphorylation was observed in cGKI-KO.

There is evidence, that TGF-β protein levels are reduced *in vivo* through RLX treatment (Garber et al., 2001). But consistent with published data from Hewitson et al. (2010), we confirmed that RLX has no influence on TGF-β1 protein levels in kidney tissue *in vivo*. Most important TGF-β effects are regulated through phosphorylation of Smad proteins (Massague, 1998) or enhanced ERK1/2 signaling (Inoki et al., 2000). Suzuki et al. (2004) as well as Piek et al. (2001) have shown, that fibronectin synthesis is regulated via the TGF-β-dependent ERK1/2-signaling pathway. Consistent with previous publications (Mookerjee et al., 2009; Chow et al., 2014; Wang et al., 2016) we found reduced Smad2 phosphorylation through RLX treatment to abrogate ECM accumulation and myofibroblast differentiation. TGF-β-dependent ERK signaling was also inhibited by reduced phosphorylation of ERK1. As no effects of reduced Smad2 or ERK1 phosphorylation were observed in the cGKI-KO, we suspect the involvement of cGKI in the suppression of TGF-β signaling through RLX. The inhibition of ERK1 phosphorylation through cGMP is in accordance to previous reports (Yeh et al., 2010; Beyer et al., 2015). However, in cultured rat renal myofibroblasts ERK1/2 phosphorylation was enhanced (Mookerjee et al., 2009; Chow et al., 2012) and, in contrary, in human renal fibroblasts no influence on ERK1/2 phosphorylation was observed via RLX treatment (Heeg et al., 2005). Species and cell-specific changes may explain different regulations.

The gelatinases MMP2 and MMP9 are ECM-degrading enzymes involved in the remodeling processes of the kidney, but their role is very complex. They are differently regulated in published work (Heeg et al., 2005; Hewitson et al., 2007, 2010; Chow et al., 2012). Upregulation increases the degradation of ECM, but on the other hand they also activate TGF-β (Abreu et al., 2002) and increase the degradation of collagen IV, which is mainly included in the basement membrane and, therefore, favors EMT (Ronco et al., 2007).

Matrix metalloproteinase 2 was strongly upregulated in fibrotic kidney, whereas MMP9 was unchanged in this pathological condition. Consistent with the current work, in rodents after UUO elevated MMP2 levels (Sharma et al., 1995) and decreased MMP9 activity (Gonzalez-Avila et al., 1998) were found. MMP2 gene expression was shown to be increased through Smad2-dependent TGF-β signaling (Piek et al., 2001). Through RLX treatment both latent and active MMP2 were decreased, which might be explained through reduced phosphorylation of Smad2. The activity of MMP9 was increased. Hewitson et al. (2010) showed similar results 9 days after UUO, where MMP2 correlated with disease severity. The regulation of MMP2 and MMP9 is assumed to be dependent on cGKI, as cGKI-KO mice had no treatment-dependent MMP regulation.

Connective tissue growth factor is an early downstream gene of TGF-β, which augments fibrotic effects by directly potentiating TGF-β effects and inhibiting the renoprotective BMP7 signaling. Furthermore it interacts with different growth factors and ECM components, to modify their functions or turnovers (Lee et al., 2015). But most of the signaling mechanisms of CTGF remain unknown. Consistent with our results, Yokoi et al. (2004) demonstrated increased CTGF levels in rats 7 days after UUO and improved kidney fibrosis through antisense oligonucleotide treatment. The reduction of CTGF protein levels is supposed to be mediated through cGKI, as no effects were observed in the cGKI-KO mice in our study. Nevertheless, data from TGF-β stimulated rat renal cortical fibroblasts showed that RLX had no influence on mRNA levels of CTGF (Masterson et al., 2004).

Kidney function was estimated through measurement of serum creatinine levels using HPLC method. In accordance with our results, Honma et al. (2014) also showed significantly increased serum levels in mice 7 days after UUO. Through the treatment of RLX a significant improvement of kidney function, estimated by decreased serum creatinine levels, was observed in our experiments as well as in previous experiments in RLX^–/–^ mice (Samuel et al., 2004) and a antiglomerular basement membrane model (McDonald et al., 2003), when treated with RLX. Whether this increase is due to increased renal plasma flow and glomerular filtration rate (Danielson et al., 2006) of the contralateral kidney or due to structural changes in the obstructed kidney remains unclear.

## Conclusion

Serelaxin signals via RXFP1 and the increase of NO/cGMP and inhibits Smad- and ERK1-dependent TGF-β signaling through cGKI. cGKI is additionally involved in PDE5a-phosphorylation for the autoregulation of cGMP-dependent effects. Our results identify RLX as antifibrotic agent and broaden the understanding of its signaling process involving cGMP/cGKI. Further elucidation of the RLX-signaling pathways will be important for its possible applicability in the treatment of CKDs.

## Author Contributions

VW, ES, LF, and JS were involved in the conception, design and interpretation of the experiments; VW, ES, FK, and JS performed and analyzed the experiments; FH contributed essential material; VW, ES, and JS wrote the manuscript; all authors were involved in the critical revision of the manuscript for important intellectual content.

## Conflict of Interest Statement

The Ph.D. thesis of VW is funded by Novartis Pharma. VW and LF are employees of Novartis Pharma. All the other authors declare that the research was conducted in the absence of any commercial or financial relationships that could be construed as a potential conflict of interest.

## Abbreviations

α-SMA: α-smooth muscle actin
cGKI: cGMP-dependent protein kinase 1
cGMP: cyclic guanosine monophosphate
CKD: chronic kidney disease
Col1A1: collagen1A1
CTGF: connective tissue growth factor
ECM: extracellular matrix
eNOS: endothelial nitric oxide synthase
ERK: extracellular-signal regulated kinase
GAPDH: glyceraldehyde-3-phosphate dehydrogenase
KO: knock out
MMP: matrix metalloproteinase
NO: nitric oxide
PDE: phosphodiesterase
P-VASP: phospho-vasodilator-stimulating phosphoprotein
RLX: serelaxin
RXFP1: relaxin family peptide receptor 1
Smad: small mothers against decapentaplegic protein
TGF-β: transforming growth factor-β
UUO: unilateral ureteral obstruction
WT: wildtype.
